# Editorial: Rocks, plants, and microbes, volume II

**DOI:** 10.3389/fpls.2023.1267003

**Published:** 2023-08-30

**Authors:** Bruno Britto Lisboa, Camille Eichelberger Granada, Luciano Kayser Vargas

**Affiliations:** ^1^ Laboratório de Química Agrícola, Departamento de Diagnóstico e Pesquisa Agropecuária (DDPA), Secretaria Estadual da Agricultura, Pecuária e Desenvolvimento Rural, Porto Alegre, Brazil; ^2^ Programa de Pós-graduação em Biotecnologia, Universidade do Vale do Taquari – Univates, Lajeado, Brazil

**Keywords:** biological weathering, crushed rocks, plant growth-promotion, soil amendment, carbon sequestration

The application of ground silicate rocks, a technique known as stonemeal, can be an alternative for soil amelioration which is gaining increasing importance in the context of the search for economically and environmentally sustainable agriculture. It is recognized that rock powders can be a source of plant nutrients, mainly by supplying P, K, Fe, and Si, as well as trace nutrients to the plants (Ribeiro et al., 2020). Rocks also have the capacity to raise the pH of the soil, acting as correctives of acidity and releasing other nutrients, such as Ca and Mg.

Besides supplying nutrients, this low-cost technology allows the so-called remineralization process, that is, the recomposition of mineralogical soil, causing the formation of chemically active secondary minerals such as smectites and illites (Theodoro et al., 2021). The alteration of the mineralogy with the presence of these minerals results in the geological rejuvenation of the soil, with an increase in its cation exchange capacity (CEC) and its fertility as a whole. Also, the addition of ground rocks that release Ca from silicates provides a way of removing carbon dioxide from the atmosphere by producing pedogenic carbonate minerals.

The process of decomposition of the primary minerals from ground rock, resulting in the release of nutrients and the formation of secondary minerals, depends on its granulometry and is mediated by the microbiota of the soil as well as the plants. The action of microorganisms leads to the solubilization of rock minerals by two main mechanisms of action: acidification of the environment, by the production of organic acids that, through exchanges of binders, replace cations of the minerals with organic cations; and the production of siderophores, chelants with high affinity for Fe and other elements that destabilize the mineral. The growth of plant roots, in turn, can produce a mechanical effect of rock disintegration besides also releasing organic acids and siderophores, such as citrate, that will promote hydrolysis reactions and the complexation of elements derived from the rock minerals. Additionally, plants exert an indirect effect on ground rocks by stimulating soil microbial activity. Thus, the rhizospheric and mycorrhizal environments are fundamental for the transformation of ground rocks and soil recovery.

The carbon dioxide removal by the dissolution of Ca-containing silicates was studied by Jariwala et al. The authors evaluated the enhanced weathering of a skarn containing wollastonite (CaSiO_3_) in association with diopside (CaMgSi_2_O_6_). Overall, amending soils with skarn improved plant yield and quality by increasing plant-available Si, as evidenced by higher Si uptake. The newly formed pedogenic calcium carbonate (CaCO_3_) was higher in the skarn-amended soils than in the control treatment, demonstrating the inorganic sequestration of CO_2_ by enhanced weathering. Carbon dioxide removal was proportional to the skarn dose and depended on the presence of active plant roots.

The formation of pedogenic calcium carbonate and the consequent CO_2_ sequestration is less likely to occur in soils with a pH below 5.2 (Dietzen and Rosing, 2023). That is not the only process affected by soil pH. It also affects soil phosphorus dynamics, including the performance of phosphate solubilizer microorganisms, as investigated by Boubekri et al. The authors assessed the interaction between the inoculation with five potential phosphate-solubilizing *Actinomycetota* (PSA) and four rock phosphates (RP) on the growth and yield of wheat in unsterilized P-deficient alkaline and acidic soils. All PSA significantly improved plant growth in treatments fertilized with RP3 and RP4. However, the combined application of *Nocardiopsis alba* BC11 along with RP4 in alkaline soil was effective in optimizing wheat yield attributes and improving the yield biomass by up to 19.7% compared to the triple superphosphate.

The negative effects of soil salinity on plants can also be attenuated by the amendment with certain rocks, as shown by An et al. A coastal silt soil (CSS) with poor permeability, high salinity, and poor nutrient levels was amended with combinations of river sand, serpentine phyllosilicate, and organic fertilizer. The salt-tolerant legume *Sesbania cannabina* was planted in the different treatments. The application of organic fertilizer combined with river sand and serpentine significantly reduced the soil moisture content and the indicators related to saline-alkali soil. The method has also greatly improved the growth conditions of *S. cannabina* and positively impacted its rhizosphere bacterial community.

Shallow rocky soils, such as the ones of karst ecosystems, different from deep and highly weathered soils, will not benefit from stonemeal. On the other hand, they will also heavily depend on microbial activity to release nutrients for plant establishment. In this scenario, arbuscular mycorrhizae fungi (AMF) play a central role by providing plant access to poorly soluble, low-diffusion nutrients, such as phosphorus, through hyphal networks (Silva et al., 2019). Shen et al. used *Bidens pilosa* L., a typical pioneer succession species, as a plant model to study how AMF regulates plant growth and nutrient utilization under patchy habitats with varying soil compositions in the karst ecosystem. The study suggested that plant growth and nutrition regulated by AMF depended more on the substrate gravel content than on the spatial patchiness in the heterogeneous karst habitat.

Thus, this Research Topic, in its second volume, brings new insights into the use of rock powders to improve soil fertility, plant productivity, and environmental quality, and shows how microbes and plants play a central role in these processes. We hope this Research Topic would stimulate new discussions and encourage new research on this subject of increasing interest and importance.

## Author contributions

BL: Writing – original draft, Writing – review & editing. CG: Writing – original draft, Writing – review & editing. LV: Writing – original draft, Writing – review & editing.
